# Developing and Testing a Patient Feedback Survey for Patients Referred to the Bromsgrove Memory Clinic for Memory Assessment

**DOI:** 10.1192/bjo.2024.522

**Published:** 2024-08-01

**Authors:** Mohammed Yousef, Richard Wooster, Lathika Weerasena

**Affiliations:** 1 Herefordshire and Worcestershire Health and Care NHS Trust, Worcester, United Kingdom; 2 Herefordshire and Worcestershire Health and Care NHS Trust, Bromsgrove, United Kingdom

## Abstract

**Aims:**

A patient feedback survey was designed based on Memory Service National Accreditation standards. The aim was to measure if the Bromsgrove Memory Clinic was meeting these standards and to show if a patient survey could be obtained in a group with cognitive issues.

**Methods:**

The 18-question survey was offered to patients referred by General Practitioners for memory assessment. The survey was designed with cognitive difficulties in mind and could be filled out jointly by patients and their accompanying person. Answers were collected using Likert scale format. 23 completed surveys were collected. Large font and facial expression diagrams were used to aid the participants. Surveys were collected during outpatient/home visits by clinicians (doctors/nurses) trained in memory assessment.

**Results:**

From the first question, 12/23 strongly agreed they received enough information prior to the appointment to feel well prepared about what to expect, 9 chose agreed and 2 gave neutral answers. The second question asked how long they waited to receive an appointment, 13 were seen within 6 weeks or less from point of referral, 7 waited between 6-weeks and 3-months. 16 participants strongly agreed to feeling happy with appointment date/time. 17 strongly agreed the clinic was easy to locate.

Next, 13/23 strongly agreed, 8 agreed, 1 was neutral that they felt confident the clinic would be able to meet their needs and 1 disagreed. Question seven asked “did staff explain the assessment process to you?” and 20/23 strongly agreed. Question eight asked if participants were given opportunity to ask questions, 22/23 strongly agreed. 21/23 strongly agreed enough time was given to discussing important information. 17/23 strongly agreed they knew they could stop the assessment at any point. 22/23 strongly agreed their privacy was respected.

Questions 12–14 looked at information given, and they could class it as “Not enough, Right amount, Too much or Not applicable”. 19/23 indicated “right amount” for information given about diagnosis. 21/23 felt information about investigations or tests was the right amount. Regarding information about medications, 20 selected “the right amount”.

18 strongly agreed the information, advice and support was helpful and sufficient. 19 strongly agreed they were treated with respect and dignity. 20 strongly agreed they wouldn't hesitate to recommend the service to others.

**Conclusion:**

The results showed an overwhelmingly positive memory service experience in Bromsgrove and it is possible to meaningfully survey this patient group with some adjustments. The survey could be repeated to monitor standards over time.

